# Influence of a School-based Physical Activity Intervention on Cortical Bone Mass Distribution: A 7-year Intervention Study

**DOI:** 10.1007/s00223-016-0174-y

**Published:** 2016-07-12

**Authors:** Jesper Fritz, Rachel L. Duckham, Timo Rantalainen, Björn E. Rosengren, Magnus K. Karlsson, Robin M. Daly

**Affiliations:** 1 Institute for Physical Activity and Nutrition, Deakin University, Geelong, Australia; 2 Clinical and Molecular Osteoporosis Research Unit, Department of Orthopedics, Institution of Clinical Research, Lund University, Skåne University Hospital, 205 02 Malmö, Sweden

**Keywords:** Bone distribution, Children, Physical activity, pQCT

## Abstract

Cortical bone mass and density varies across a bones length and cross section, and may be influenced by physical activity. This study evaluated the long-term effects of a pediatric school-based physical activity intervention on tibial cortical bone mass distribution. A total of 170 children (72 girls and 98 boys) from one school were provided with 200 min of physical education per week. Three other schools (44 girls and 47 boys) continued with the standard 60 min per week. Tibial total and cortical area, cortical density, polar stress–strain index (SSI), and the mass and density distribution around the center of mass (polar distribution, mg) and through the bones cortex (radial distribution subdivided into endo-, mid-, and pericortical volumetric BMD: mg/cm^3^) at three sites (14, 38, and 66 %) were assessed using peripheral quantitative computed tomography after 7 years. Girls in the intervention group had 2.5 % greater cortical thickness and 6.9 % greater SSI at the 66 % tibia, which was accompanied by significantly greater pericortical volumetric BMD compared to controls (all *P* < 0.05). Region-specific differences in cortical mass were also detected in the anterior, medial, and lateral sectors at the 38 and 66 % tibial sites. There were no group differences at the 14 % tibia site in girls, and no group differences in any of the bone parameters in boys. Additional school-based physical education over seven years was associated with greater tibial structure, strength, and region-specific adaptations in cortical bone mass and density distribution in girls, but not in boys.

## Introduction

Regular participation in moderate to high impact sports (e.g., gymnastics, basketball, volleyball) during growth has been associated with greater bone mass, structure, and strength [1–3], traits associated with a reduced risk of fracture [4]. There is also some evidence that these traits can be improved through school-based physical activity programs that incorporate targeted weight-bearing activities (e.g., jumping, hopping, skipping) [5–7], with the greatest benefits apparent during the pre- and early pubertal period [2, 7, 8]. However, there are few long-term intervention studies [9, 10] examining the effects of more generalized, school-based physical education (PE) programs on cortical bone structure and the mass/density distribution during growth. This is important because increased loading can induce small localized cortical bone adaptations to resist fractures at sites subjected to the greatest loads (strains) that are not necessarily detected using techniques that simply measure whole cortical bone mass or structure [11].

It is well established that the mass and density of cortical bone varies across its cross section and along its axial length, and that interindividual differences likely reflect adaptations that occur in response to increased (or decreased) loading [12, 13]. During growth, bone modeling is the primary factor associated with exercise-induced changes in cortical bone geometry and mass distribution around the center of mass or neutral axis (polar distribution) [14]. In contrast, any variation in cortical density and its circumferential distribution (radial distribution) are likely to be related to changes in intracortical remodeling that alter the porosity and/or mineralization of bone [15, 16]. While a number of cross-sectional studies in children, adolescents, and young adults and a longitudinal intervention by MacDonald et al. [10] have reported that higher level of physical activity or participating in high- or odd-impact sports or weight-bearing exercises is associated with localized increases in cortical bone mass (e.g., an increase in bone mass in the anterior-posterior region of the tibia which is the primary direction of bending in response to loading at the midtibial shaft) [10, 16–18], no study has primarily investigated the effects of a long-term school-based physical activity intervention on cortical bone mass distribution.

This study is a secondary analysis of a 7-year school-based PE intervention in which we have previously reported that girls engaged in the intervention experienced greater gains in total body and spine bone mineral density (BMD) and had greater tibial cortical thickness at the 66 % site, while no beneficial effects were detected in boys [7]. Building upon these findings, the aim of this study was to evaluate the effects of the 7-year school-based PE intervention on both polar cortical mass distribution and radial volumetric BMD distribution along the tibial cortex in girls and boys, since these effects may be present with or without the effect on other bone parameters.

## Materials and Methods

### Study Design

The Malmo pediatric osteoporosis prevention (POP) study is a prospective controlled physical activity intervention study designed to examine the effects of increased school-based PE on various health outcomes in Swedish children. More specific details about the study design have been reported previously [19, 20]. Briefly, four government-funded elementary schools within the same geographic area and with a similar socioeconomic and ethnic structure agreed to participate. One of the schools was invited to be the intervention school, while the remaining three served as control schools. As previously reported [7], there was low variability in various lifestyle, anthropometric, and bone parameters between the schools.

### Participants

**Fig. 1:**
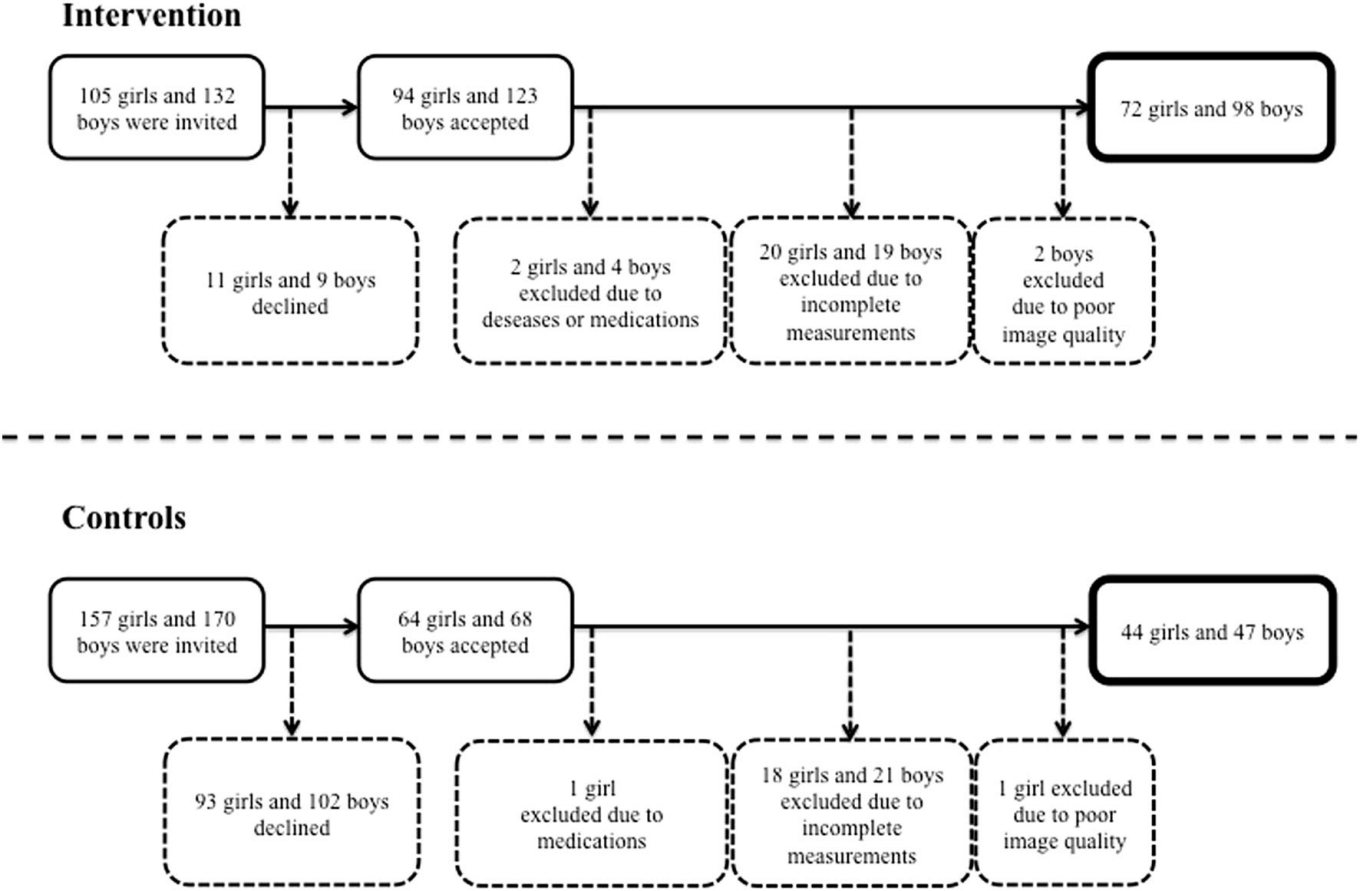
Flow chart describing number of participants included and drop outs

Prior to any intervention, all children with a school start year in 1998–2000 in the intervention school and 1999–2000 in the control schools were invited to enroll in the study. At the initial baseline testing, all children were aged between 6 and 9 years. As reported previously [7, 19], in the intervention group 94 of the 105 invited girls and 123 of the 132 invited boys agreed to participate. For the current analyses involving the 7-year follow-up, we excluded two girls and four boys in the intervention group due to diseases or medications that could affect growth, bone health, or muscle development. A further 20 girls and 19 boys were excluded due to incomplete baseline or follow-up measurements and two more boys were excluded due to poor image quality. In the control schools, a total of 64 of the 157 invited girls and 68 of the 170 invited boys agreed to participate in the study. For the current analyses, one girl was excluded due to medication that could affect growth, 18 girls and 21 boys due to incomplete baseline or follow-up measurements, and one girl due to poor image quality. This resulted in a final cohort of 72 girls and 98 boys in the intervention group and 44 girls and 47 boys in the control group (Fig. 1). Dropout analyses demonstrated similar baseline measurements in terms of age, height, and weight between those children who only attended baseline and those who also attended the 7-year follow-up (data not shown). Also, there were no differences in anthropometric characteristics (based on the first grade compulsory school health examinations) for those who declined to participate in the study and those who accepted [21].

### Intervention

As reported previously [19, 20], PE is a compulsory school subject in Sweden and children participate in 60 min per week of PE. While the children in the three control schools continued with the compulsory standard of 60 min per week of PE, the amount of PE in the intervention school was increased to 200 min per week which was administered in a 40-min lesson every school day throughout the 7-year study period. All PE classes were conducted by the regular teachers according to the regular PE curriculum and included activities such as ball games, running, and jumping. There were no extra PE classes during weekends or school holidays.

### Lifestyle, Health, and Physical Activity

Together with parents or guardians, the children answered a questionnaire, previously used in several studies but modified for use in children [22–24], to evaluate lifestyle factors such as use of medications, diseases, nutrition, smoking, menarche, and spare-time physical activity both at baseline and at follow-up. Total physical activity was calculated as regular organized spare-time PA plus school curriculum PA. A school nurse assessed pubertal maturation according to Tanner stage at the start of the study and the children self-assessed tanner stage at follow-up. Height and weight were measured using standard equipment both at baseline and at follow-up.

### Bone Mass, Structure, and Distribution

The main outcomes in this study, regional distribution of bone mass and structure, were assessed only at follow-up. We have previously reported cortical density, cortical thickness, and total area for the 66 % tibial site [7]. Thus, the regional distribution data in this study are cross-sectional data, even though the POP study is commonly referred to as a prospective controlled intervention study. Cortical bone structure and density parameters, estimates of bone strength, and regional body composition of the left lower limb were assessed using peripheral quantitative computed tomography (pQCT, XCT 2000^®^, Stratec, Pforzheim, Germany). For all participants, a left leg scout scan was performed to determine the 14, 38, and 66 % of the tibia length from the distal end of the medial malleolus, after which each site was scanned. The voxel size was set to 0.5 mm, the slice thickness to 2 mm, and the scanning speed to 20 mm/s. The manufacturer's software package (Stratec Medical, Pforzheim, Germany, version 6) in conjunction with edge detection and thresholding steps was used to acquire densitometric and structural parameters of bone and soft tissue. The periosteal surface of the tibia diaphysis was found by using a contour algorithm based on a threshold of 280 mg/cm^3^ from which total bone area (mm^2^) was estimated. The polar SSI (mm^3^) was derived to estimate diaphyseal bone resistance to torsional loading [25]. As reported previously [16], cortical bone was selected by thresholding at 550 mg/cm^3^ for the 14 % site and 710 mg/cm^3^ for the 38 and 66 % sites, from which cortical area (mm^2^), cortical density (mg/cm^3^), and cortical thickness (mm) were derived. Subcutaneous fat cross-sectional area (CSA, cm^2^) was obtained by selecting the area with thresholds −40–40 mg/cm^3^ hydroxyapatite density. Muscle CSA (cm^2^) was obtained by subtracting fat CSA and total bone area from the CSA of the total limb. The coefficient of variation (CV %), evaluated by duplicate measurements in our lab in 13 healthy children, was 0.5 % for cortical volumetric BMD (vBMD).

**Fig. 2:**
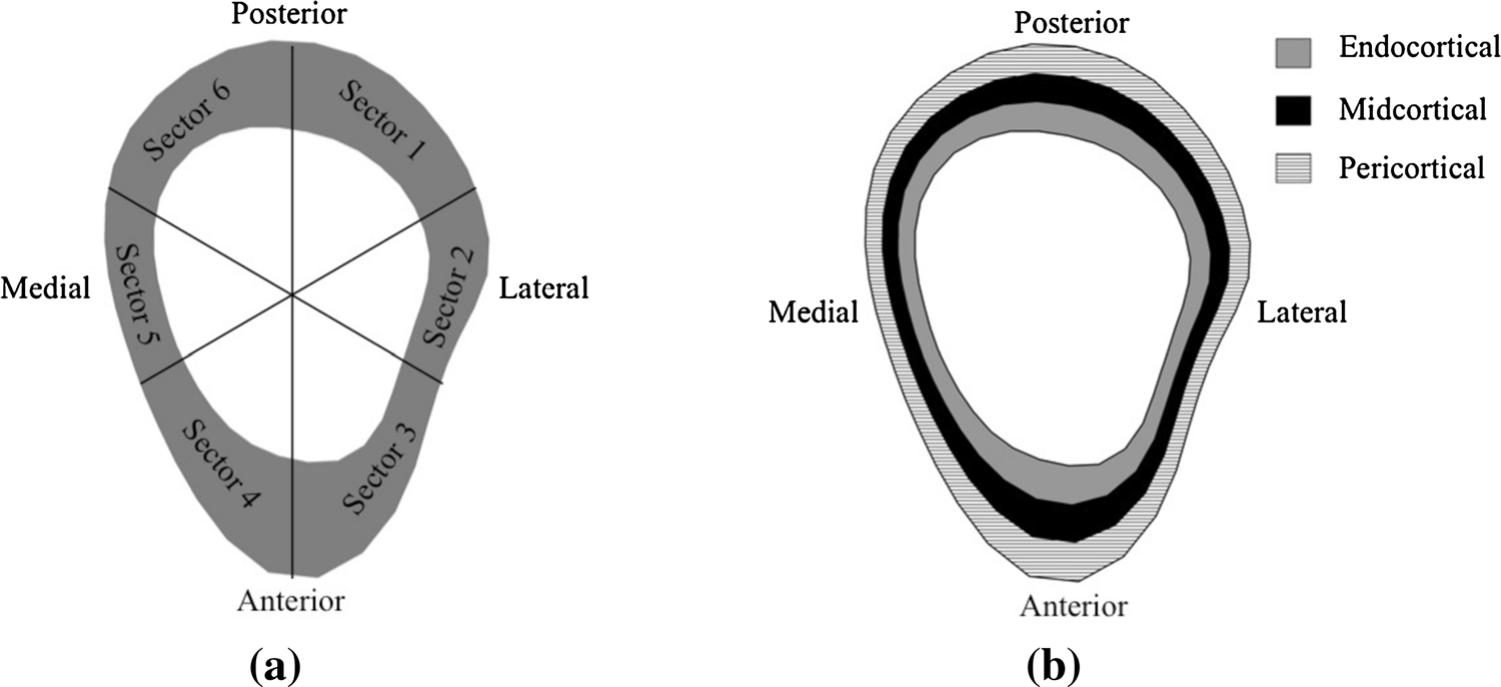
Example of the polar (**a**) and radial (**b**) distribution analysis of the left tibia

Polar distribution (cortical bone mineral mass, mg) and radial distribution (radial vBMD, mg/cm^3^) of the tibia were estimated using Image J as described previously [11]. Briefly, a threshold of 550 mg/cm^3^ for the 14 % site and 710 mg/cm^3^ for the 38 and 66 % sites with a 3 × 3 median filtering of the image was used to differentiate the cortical bone from the surrounding soft tissue and bone marrow. To eliminate partial volume effects, the outermost and innermost layers of cortical pixels were excluded from the analysis. We aligned the bones between individuals according to tibia and fibula marrow center and calculated polar distribution by subdividing the tibia cortex into six sectors around its center of mass with the average bone mass estimated for each sector (Fig. 2a). Radial distribution was estimated by subdividing the cortex into three concentric rings (endocortical, midcortical, and pericortical) by first removing all pixels below the threshold of each site from the image and subdividing the remaining cortical bone into three concentric circles with the same thickness. The thickness of the rings varied around the cortex according to the anatomy. The innermost ring is referred to as endocortical, the midring as midcortical, and the outermost ring as the pericortical (Fig. 2b).

### Statistical Analysis

All statistical analyses were performed with IBM SPSS Statistics^®^ version 20. Results are reported as absolute numbers, means with standard deviations (SD), or means with 95 % confidence intervals (95 % CI), unless otherwise stated. We tested group differences by Student’s *t* test between means, Fisher’s exact test, Pearson’s Chi-square test, or Mann–Whitney U-test. For bone distribution parameter comparisons between groups at follow-up, we used analysis of covariance (ANCOVA), adjusting for age at baseline. In a second model, we used ANCOVA adjusted for age at baseline, Tanner stage at follow-up, weight at follow-up, and tibial length at follow-up. *P* < 0.05 was regarded as statistically significant.

## Results

Characteristics of boys and girls in intervention and control groups are shown in Table 1. At baseline, girls and boys in the control groups were on average 0.3 years older than their intervention counterparts, and a higher proportion of boys in the intervention group excluded dairy products from their diet compared to controls (Table 1). At follow-up, girls in the intervention group had longer tibia and a larger proportion indicated some form of medical condition compared to controls, while boys in the intervention group had smaller muscle CSA at the tibial 38 % site than male controls (Table 1).

**Table 1:** Physical characteristics and lifestyle factors at baseline and at the 7-year follow-up and tibia composition at 14, 38, and 66 % at follow-up in girls and boys in the intervention and control groups

	Girls			Boys		
	Intervention	Control	*P* value	Intervention	Control	*P* value
	*n* = 72	*n* = 44		*n* = 98	*n* = 47	
Baseline						
Characteristics						
Age (years)	7.5 ± 0.5	7.8 ± 0.6	**0.004**	7.6 ± 0.6	7.9 ± 0.6	**0.001**
Height (cm)	127.3 ± 5.5	127.8 ± 7.3	0.715	128.3 ± 6.6	129.9 ± 6.0	0.169
Weight (kg)	27.1 ± 5.4	26.7 ± 5.2	0.715	27.5 ± 5.4	28.0 ± 4.9	0.641
BMI (kg/m^2^)	16.6 ± 2.7	16.2 ± 1.8	0.323	16.6 ± 2.4	16.5 ± 1.9	0.745
Tanner stage (1/2/3/4/5), (*n*)	72/0/0/0/0	44/0/0/0/0	NA	98/0/0/0/0	47/0/0/0/0	NA
	(100/0/0/0/0 %)	(100/0/0/0/0 %)		(100/0/0/0/0 %)	(100/0/0/0/0 %)	
Lifestyle factors						
Excluding dairy (*n*)	1 (1 %)	1 (2 %)	0.697	0 (0 %)	6 (13 %)	**<0.001**
Current disease (*n*)	6 (9 %)	3 (8 %)	0.827	12 (13 %)	3 (7 %)	0.278
Current medication (*n*)	8 (12 %)	2 (5 %)	0.250	16 (17 %)	4 (9 %)	0.201
Follow-up						
Characteristics						
Age (years)	14.2 ± 0.8	14.3 ± 1.0	0.449	14.4 ± 0.7	14.4 ± 1.0	0.732
Height (cm)	164.8 ± 6.1	163.0 ± 7.4	0.153	170.3 ± 9.5	170.0 ± 10.0	0.849
Weight (kg)	56.2 ± 11.7	52.5 ± 9.5	0.077	59.2 ± 14.1	58.0 ± 12.4	0.613
BMI (kg/m^2^)	20.6 ± 3.8	19.6 ± 2.5	0.098	20.2 ± 3.6	19.9 ± 3.2	0.640
Tanner stage (1/2/3/4/5), (*n*)	0/2/12/35/23	0/0/8/23/13	0.955	0/5/12/28/53	0/0/8/17/22	0.594
	(0/3/17/49/32 %)	(0/0/18/52/30 %)		(0/5/12/29/54 %)	(0/0/17/36/47 %)	
Lifestyle factors						
Excluding dairy (*n*)	1 (2 %)	2 (6 %)	0.552	1 (1 %)	1 (3 %)	0.521
Current disease (*n*)	9 (16 %)	0 (0 %)	**0.016**	8 (12 %)	3 (10 %)	0.781
Current medication (*n*)	9 (17 %)	1 (3 %)	0.064	13 (19 %)	2 (7 %)	0.109
Smoker (*n*)	0 (0 %)	2 (5 %)	0.068	2 (2 %)	0 (0 %)	0.324
Alcohol consumer (*n*)	5 (7 %)	1 (2 %)	0.270	12 (12 %)	2 (4 %)	0.127
Menarche	54 (75 %)	31 (70 %)	0.591	NA	NA	NA
Tibia composition*						
Tibia length (mm)	364 ± 23	355 ± 30	**0.048**	372 ± 30	377 ± 31	0.221
Tibia muscle CSA 14 % (cm^2^)	16.6 ± 2.9	16.1 ± 2.1	0.289	19.0 ± 3.3	19.8 ± 3.3	0.088
Tibia muscle CSA 38 % (cm^2^)	31.7 ± 6.7	30.4 ± 4.9	0.172	33.6 ± 7.3	35.8 ± 6.4	**0.046**
Tibia muscle CSA 66 % (cm^2^)	55.3 ± 10.5	52.4 ± 6.8	0.073	61.3 ± 11.1	61.9 ± 10.3	0.621
Tibia fat CSA 14 % (cm^2^)	16.5 ± 5.9	14.9 ± 4.3	0.106	12.0 ± 5.3	12.1 ± 4.3	0.916
Tibia fat CSA 38 % (cm^2^)	24.9 ± 8.4	23.1 ± 6.1	0.201	29.4 ± 6.9	19.5 ± 5.5	0.920
Tibia fat CSA 66 % (cm^2^)	28.0 ± 10.3	25.3 ± 7.0	0.123	20.8 ± 8.6	20.3 ± 6.5	0.725

Data are presented as means ± standard deviations or as number of children with proportions within brackets. Statistically significant group differences are bolded

*NA* non applicable

* Analyses adjusted for age at follow-up

Prior to the intervention, the amount of organized physical activity was similar in each of the gender-specific intervention groups and their respective controls. In contrast, after the initiation of the intervention and during the entire 7-year study period, the total amount of physical activity was on average 1.9–3.2 h per week higher in the girls and 2.0–3.1 h per week higher in the boys in the intervention compared to the control group (Table 2).

**Table 2:** Duration of physical activity (PA) just before study start, just after study start, after 4 and 7 years in girls and boys in the intervention and control groups

	Girls			Boys		
	Intervention	Control	*P* value	Intervention	Control	*P* value
	*n* = 72	*n* = 44		*n* = 98	*n* = 47	
PA before study start (hours/week)						
Total PA	1.7 ± 1.7	2.2 ± 2.0	0.198	3.1 ± 3.5	3.4 ± 3.2	0.635
PA after study start (hours/week)						
School curriculum	3.3	1.0	NA	3.3	1.0	NA
Outside school	1.7 ± 1.7	2.2 ± 2.0	0.198	3.1 ± 3.5	3.4 ± 3.2	0.635
Total PA	5.1 ± 1.7	3.2 ± 2.0	**<0.001**	6.4 ± 3.5	4.4 ± 3.2	**0.001**
PA 4 years after study start (hours/week)						
School curriculum	3.3	1.0	NA	3.3	1.0	NA
Outside school	4.5 ± 3.1	4.5 ± 3.5	0.976	6.1 ± 4.5	5.3 ± 3.2	0.285
Total PA	7.8 ± 3.1	5.5 ± 3.5	**<0.001**	9.4 ± 4.5	6.3 ± 3.2	**<0.001**
PA at 7-year follow-up (hours/week)						
School curriculum	3.3	1.0	NA	3.3	1.0	NA
Outside school	5.0 ± 3.3	4.1 ± 3.2	0.163	6.4 ± 4.8	5.7 ± 4.1	0.404
Total PA	8.3 ± 3.3	5.1 ± 3.2	**<0.001**	9.7 ± 4.8	6.7 ± 4.1	**<0.001**

Data are presented as means ± standard deviations. Statistically significant differences are bolded

*NA* non applicable

**Fig. 3:**
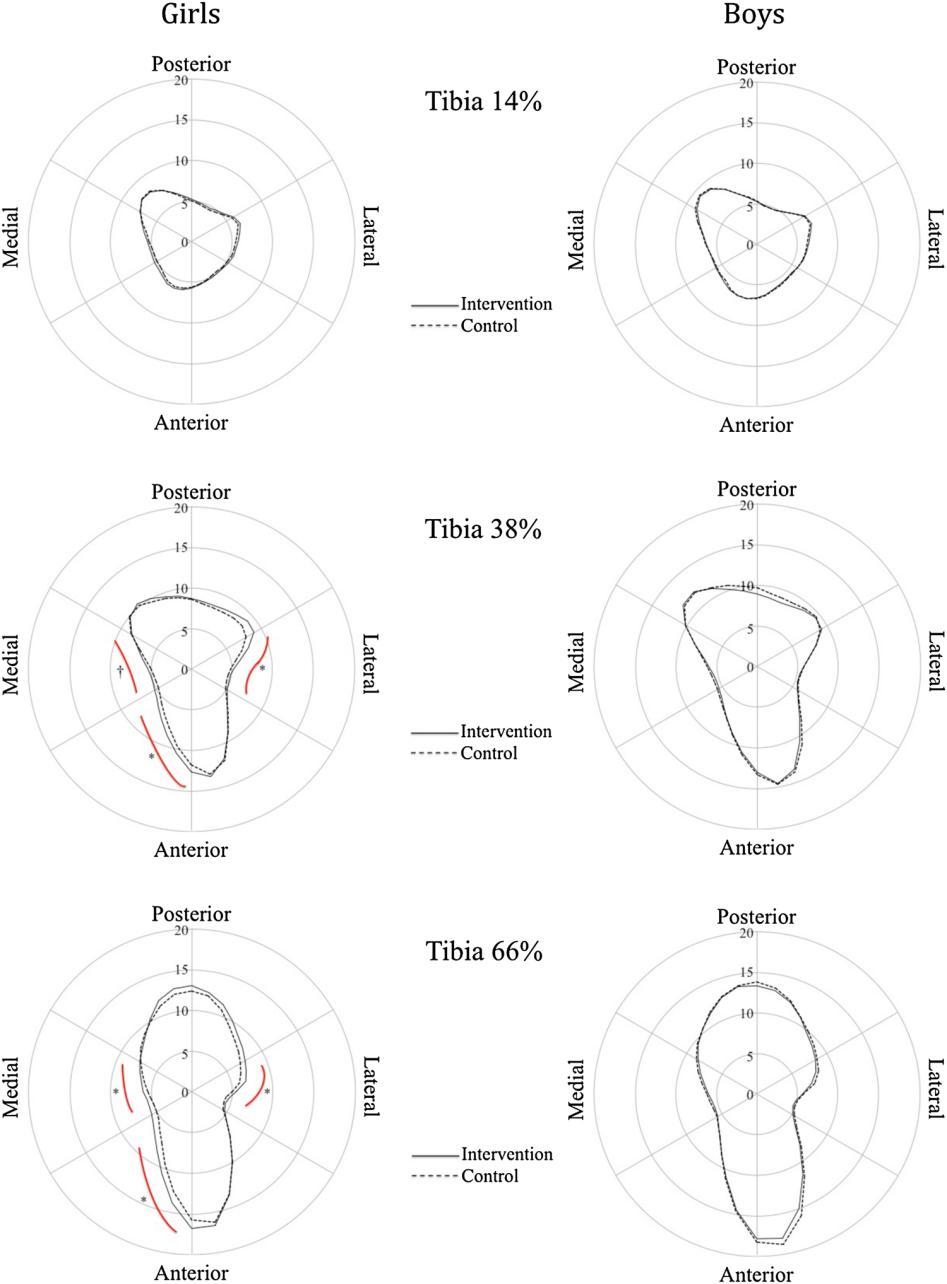
Mean age-adjusted cortical bone mineral mass (mg) at tibia 14, 38, and 66 % at the 7-year follow-up in the boys and girls in the intervention compared to control group; *dagger* represents *P* < 0.05 and *asterisk* represents *P* < 0.01

As previously reported, girls in the intervention group had greater cortical thickness at the 66 % tibia at the 7-year follow-up [7]. At the 7-year follow-up, girls in the intervention group also had greater SSI at the 66 % tibia compared to controls (*P* < 0.05), which was accompanied by greater mineral mass in the lateral [+0.6 mg (95 % CI 0.2, 1.0)], anterior-medial [+1.1 mg (0.4, 1.9)], and medial [+0.6 mg (0.2, 0.9)] sectors and greater radial vBMD in the pericortical region [+11.1 mg/cm^3^ (0.2, 22.0)] compared to controls (*P* ranging from <0.05 to <0.001). At the 38 % tibia site, bone mass distribution analysis revealed that girls in the intervention group had significantly greater mineral mass in the lateral [+0.6 mg (0.3, 1.0)], anterior-medial [+0.8 mg (0.3, 1.4)], and medial [+0.4 mg (0.1, 0.8)] sectors compared to controls (*P* ranging from <0.05 to <0.001). When expressed as a percentage difference relative to controls, the adjusted differences in favor of the intervention group was 6.9 % for SSI at the 66 % tibia, 7.7–11.7 % for mineral mass across the various sectors at each site, and 0.7 % for the pericortical vBMD at the 66 % tibia. There were no significant differences at the 14 % site in girls, and no skeletal differences in boys between the groups at any site (14, 38, or 66 %) (Table 3; Fig. 3). Using the second adjustment model, adjusting for age at baseline and Tanner stage, weight and tibial length at follow-up, the results remained largely unchanged, with the exception that the benefits of the intervention in girls at the anterior-medial sector (*P* = 0.06) and the medial sector (*P* = 0.12) at the 38 % tibial site were no longer significant.

**Table 3:** Tibia bone structure, density, and distribution (pQCT) at 14, 38, and 66 % at the 7-year follow-up in boys and girls in the intervention and control groups

	Girls			Boys		
	Intervention	Control	*P* value	Intervention	Control	*P* value
	*n* = 72	*n* = 44		*n* = 98	*n* = 47	
Tibia 14 %						
Cortical bone traits						
Cortical density (mg/cm^3^)	1039 ± 40	1036 ± 41	0.392	989 ± 46	999 ± 40	0.135
Cortical area (mm^2^)	147 ± 24	147 ± 22	0.780	167 ± 30	170 ± 30	0.351
Cortical thickness (mm)	3.8 ± 0.3	3.8 ± 0.3	0.274	4.1 ± 0.3	4.0 ± 0.3	0.699
Total area (mm^2^)	425 ± 69	413 ± 65	0.269	486 ± 74	481 ± 77	0.740
SSI polar (mm^3^)	1041 ± 237	1015 ± 212	0.320	1191 ± 273	1219 ± 286	0.412
Cortical bone mineral mass						
S1—posterior-lateral (mg)	4.9 ± 0.9	4.7 ± 0.6	0.076	5.0 ± 0.8	5.0 ± 1.0	0.788
S2—lateral (mg)	5.8 ± 1.0	5.6 ± 0.7	0.083	6.6 ± 1.1	6.4 ± 1.3	0.633
S3—anterior-lateral (mg)	5.2 ± 1.0	5.0 ± 0.6	0.163	5.8 ± 1.1	5.9 ± 1.1	0.401
S4—anterior-medial (mg)	5.8 ± 1.0	5.6 ± 0.7	0.079	6.5 ± 1.3	6.4 ± 1.3	1.000
S5—medial (mg)	5.5 ± 1.1	5.3 ± 0.7	0.118	6.3 ± 1.2	6.3 ± 1.4	0.659
S6—posterior-medial (mg)	7.0 ± 1.2	7.0 ± 0.8	0.553	7.7 ± 1.4	7.8 ± 1.5	0.461
Radial vBMD distribution						
Endocortical (mg/cm^3^)	1041 ± 43	1043 ± 50	0.924	991 ± 49	994 ± 41	0.614
Midcortical (mg/cm^3^)	1127 ± 46	1124 ± 46	0.338	1066 ± 50	1071 ± 45	0.375
Pericortical (mg/cm^3^)	1127 ± 52	1120 ± 50	0.138	1058 ± 59	1065 ± 53	0.253
Tibia 38 %						
Cortical bone traits						
Cortical density (mg/cm^3^)	1088 ± 33	1081 ± 27	0.075	1029 ± 45	1031 ± 36	0.625
Cortical area (mm^2^)	245 ± 36	235 ± 33	0.087	277 ± 47	285 ± 49	0.206
Cortical thickness (mm)	3.4 ± 0.2	3.4 ± 0.2	0.101	3.6 ± 0.3	3.7 ± 0.3	0.427
Total area (mm^2^)	368 ± 45	357 ± 47	0.169	417 ± 60	424 ± 65	0.427
SSI polar (mm^3^)	1251 ± 243	1179 ± 237	0.051	1408 ± 322	1464 ± 326	0.209
Cortical bone mineral mass						
S1—posterior-lateral (mg)	8.5 ± 1.4	8.1 ± 1.4	0.084	8.8 ± 1.6	9.2 ± 2.2	0.069
S2—lateral (mg)	6.3 ± 1.0	5.7 ± 1.1	**0.001**	6.6 ± 1.2	6.6 ± 1.3	0.875
S3—anterior-lateral (mg)	8.7 ± 1.9	8.6 ± 1.6	0.634	9.8 ± 2.0	10.0 ± 2.2	0.424
S4—anterior-medial (mg)	8.5 ± 1.5	7.8 ± 1.4	**0.005**	8.6 ± 1.8	8.8 ± 1.7	0.334
S5—medial (mg)	5.6 ± 1.0	5.2 ± 0.9	**0.023**	6.1 ± 1.1	6.3 ± 1.4	0.248
S6—posterior-medial (mg)	9.5 ± 1.9	9.3 ± 1.6	0.416	10.7 ± 2.2	10.9 ± 2.4	0.600
Radial vBMD distribution						
Endocortical (mg/cm^3^)	1113 ± 29	1107 ± 25	0.109	1059 ± 39	1056 ± 37	0.746
Midcortical (mg/cm^3^)	1145 ± 30	1142 ± 24	0.353	1086 ± 41	1086 ± 37	0.943
Pericortical (mg/cm^3^)	1148 ± 37	1142 ± 34	0.137	1075 ± 53	1076 ± 46	0.801
Tibia 66 %						
Cortical bone traits						
Cortical density (mg/cm^3^)^a^	1051 ± 30	1048 ± 29	0.300	1003 ± 35	1001 ± 32	0.936
Cortical area (mm^2^)	260 ± 38	250 ± 33	0.098	290 ± 52	290 ± 47	0.900
Cortical thickness (mm)^a^	4.1 ± 0.3	4.0 ± 0.4	**0.044**	4.4 ± 0.3	4.4 ± 0.4	0.264
Total area (mm^2^)^a^	534 ± 75	516 ± 88	0.121	603 ± 98	627 ± 113	0.122
SSI polar (mm^3^)	1787 ± 361	1672 ± 360	**0.036**	2012 ± 485	2098 ± 493	0.179
Cortical bone mineral mass						
S1—posterior-lateral (mg)	10.5 ± 1.9	9.9 ± 1.9	0.058	11.2 ± 2.2	11.4 ± 2.4	0.711
S2—lateral (mg)	6.0 ± 1.1	5.4 ± 1.1	**0.007**	6.3 ± 1.3	6.6 ± 1.2	0.337
S3—anterior-lateral (mg)	9.6 ± 1.8	9.5 ± 1.6	0.521	10.6 ± 2.3	11.1 ± 2.2	0.310
S4—anterior-medial (mg)	10.5 ± 1.9	9.4 ± 1.9	**0.002**	10.9 ± 2.3	11.0 ± 2.0	0.988
S5—medial (mg)	5.9 ± 0.8	5.4 ± 1.0	**0.001**	6.1 ± 1.1	6.3 ± 1.1	0.400
S6—posterior-medial (mg)	9.8 ± 2.0	9.6 ± 1.3	0.356	10.9 ± 2.3	11.0 ± 1.8	0.926
Radial vBMD distribution						
Endocortical (mg/cm^3^)	1083 ± 25	1084 ± 27	0.744	1037 ± 31	1042 ± 28	0.515
Midcortical (mg/cm^3^)	1121 ± 27	1117 ± 28	0.183	1067 ± 36	1071 ± 31	0.656
Pericortical (mg/cm^3^)	1125 ± 34	1117 ± 31	**0.046**	1060 ± 43	1064 ± 36	0.645

Analyses adjusted for age. Data are presented as mean ± SD. Statistically significant differences are bolded

^a^Data previously reported [7]

## Discussion

The main finding from this 7-year population-based school PE intervention which included 40 min per day of moderate physical activity initiated during the prepubertal period was that girls, but not boys, in the intervention group had greater cortical strength at the 66 % and to a lesser extent the 38 % tibia, which was accompanied by small but significant region-specific bone mass adaptations in the anterior, lateral, and anterior-medial cortices and an increase in pericortical volumetric BMD at the 66 % site. There was no effect of the intervention at the 14 % tibia site. These results suggest that a generalized, moderate-intensity school-based physical activity program can enhance bone structure and induce region-specific cortical bone adaptations throughout the tibia in girls.

While there are reports that exercise during the pre- and peripubertal years can enhance bone mass, structure, and strength at loaded skeletal sites [5, 26], there are mixed findings from the limited number of school-based exercise intervention studies that have used pQCT to evaluate exercise-induced changes in bone structure and strength [9, 10, 27–30]. This may be related, at least in part, to the relatively short follow-up periods (8–16 months) in many of these studies, which may have limited the ability to detect subtle early exercise-induced bone structural changes over and above those associated with normal growth. A 4-year school-based, specialist-led PE intervention study in prepubertal boys and girls reported that girls receiving two 50-minute sessions of specialist-led PE per week had 5.0–7.5 % greater gains in tibia 66 % cortical area and thickness compared to girls receiving common practice PE; boys receiving the specialist PE also had greater gains (5.2 %) in cortical thickness [27]. Consistent with these findings, the results from our 7-year population-based school PE intervention showed that girls receiving the intervention had 6.9 % greater cortical strength (SSI) at the 66 % tibia compared to controls. In boys, however, we found no positive skeletal benefits of the extra PE, which could be due to the gender difference in activity level with boys being more active than girls in our study, and thus less likely to experience further skeletal adaptation with the additional PE. Although cause-and-effect cannot be inferred from our study because pQCT measures of bone structure and strength were not assessed at baseline, the results provide some additional evidence that long-term school-based physical activity interventions can induce cortical bone structural changes during the pre- and peripubertal years, especially in girls.

A novel finding from our study was that the moderate-intensity school-based PE intervention was associated with region-specific cortical bone adaptations at various sites along the tibia. Specifically, we found that girls receiving the intervention had 7.7–11.7 % higher mineral mass at the lateral, anterior, and anterior-medial sectors of both the 38 and 66 % tibial sites. The results from a previous 16-month school-based physical activity intervention in boys also reported that the enhanced tibial bone strength associated the intervention was predominantly due to greater cortical area/thickness gains in the anterior, medial, and posterior quadrants of the midtibia [10]. However, in that study the between-group differences were modest (1.0–1.4 %) and not significantly different from the growth-related changes in the controls, which may be explained by the relatively short intervention period. Nevertheless, several previous cross-sectional studies in children and adolescents [16, 18] and young athletes [17] have reported that higher levels of weight-bearing impact activities or sports were associated with localized cortical adaptations, particularly in the anterior-posterior plane of the mid- and proximal tibia. This is in line with other evidence that suggest that the midtibial shafts primary loading mode is bending in anterior-posterior plane [31]. However, the result in our study that there were also region-specific adaptations in the medial and lateral planes might be explained by the fact that our generalized PE intervention included a wide range of activities, such as ball games, running, jumping, and playing, that could have created bending forces in the medial and lateral planes of the tibia.

An interesting result in our study was that there were no exercise-induced cortical bone adaptations at the 14 % tibia. Previous unilateral loading studies in young tennis players have found that the response of cortical bone to loading is site (and surface) specific [32, 33]. It is possible that these findings may relate to the hypothesis that the strain threshold for bone adaptation may vary within (and between) bones depending on the local strain environment [34, 35]. For example, a study of rat ulna found that the strain threshold to induce an osteogenic response varied along the length of the bone, with the greatest response occurring distally where strains are typically higher compared to proximally [34]. In our study, we did not observe any region-specific cortical bone adaptations more distally (14 % tibia) which may be explained by the fact that this site experiences predominantly compressive loads (strains) which may be more uniformly distributed throughout the cortex, and thus less likely to stimulate localized bone (periosteal) adaptations [36].

Several previous cross-sectional studies in children [16, 18] and young athletes [17, 37] as well as school-based physical activity interventions [9, 27–29] have reported no change, an increase or a decrease with regard to the effects of physical activity or weight-bearing impact exercise on pQCT-derived measures of cortical vBMD at the tibia. In our study, we observed no effects of the intervention on whole bone tibial cortical vBMD or radial density distribution, in which we subdivided the tibia into inner, mid-, and outer circumferential layers, with exception of a small 0.7 % higher pericortical vBMD in girls in the intervention compared to control group. Since the assessment of cortical vBMD represents an apparent mineral density that reflects a combination of intracortical porosity, mineralization, and other bone material properties [38], this may suggest that our moderate-intensity school-based PE intervention had no effect on the porosity and/or mineralization of cortical bone along the shaft of the tibia.

Consistent with the findings from our study, previous school-based intervention studies have reported mixed findings regarding the effects of physical activity on pQCT-measured bone parameters in boys compared to girls [9, 27, 28]. The finding that there was no effect of the intervention on any bone parameters among boys in our study could be due to the fact that the girls were generally less active in their spare-time prior to the commencement of the study, and thus the extra school-based physical activity which included a diverse range of weight-bearing activities was sufficient to elicit positive skeletal adaptations. Indeed, boys in our study were already undertaking a mean of >3 h per week of physical activity prior to the start of the intervention, and hence, the additional physical activity was not enough to elicit further bone adaptation. Although the optimal dose of physical activity for enhancing bone health during growth remains unknown, there is some evidence that duration of activity is a strong predictor of bone structure and estimates of bone strength at the tibia in children aged 8–13 years [39]. Furthermore, since maturational stage differs from chronological age between genders [40], even if the girls and boys are the same age and Tanner stage, they might be in different maturational stages when it comes to bone development and that could explain the gender difference in outcome.

The strengths of this study include the population-based design, the general school-based PE program which was at a level enabling all children to participate, the long-term intervention (this is the longest of its kind), and the unique technique used to quantify cortical mass and density distribution. However, there are a number of limitations. The single pQCT assessment, which is inherently vulnerable to uncontrolled factors such as reverse causality, is a limitation. Nevertheless, the primary findings in this study were independent of age, Tanner stage, bone length, and weight, which increase the likelihood that the intervention contributed to the positive skeletal adaptation rather than selection bias based on body size. Another limitation is that self-assessment in tanner staging has by some researcher been recognized as a less reliable indicator for maturity [41, 42] while others have inferred that this estimate is useful when evaluating maturity in children on group level [43, 44]. It would also have been advantageous to register participation rates and activity levels during each physical education (PE) class to better estimate overall activity levels, and include objective measures of physical activity (e.g., accelerometers) to quantify the level of loading associated with the school PE program. However, PE is a compulsory school subject in Sweden and none of the participating children failed their grade.

In conclusion, this study indicates that a 7-year moderately intense school-based physical activity intervention initiated in the prepubertal period was associated with higher tibial cortical bone strength that was accompanied by region-specific gains in cortical bone mass distribution in girls, but not in boys.

## References

[1] Gunter K, Baxter-Jones AD, Mirwald RL, Almstedt H, Fuchs RK, Durski S, Snow C (2008). Impact exercise increases BMC during growth: an 8-year longitudinal study. J Bone Miner Res.

[2] Heinonen A, Oja P, Kannus P, Sievanen H, Haapasalo H, Manttari A, Vuori I (1995). Bone mineral density in female athletes representing sports with different loading characteristics of the skeleton. Bone.

[3] Gunter K, Baxter-Jones AD, Mirwald RL, Almstedt H, Fuller A, Durski S, Snow C (2008). Jump starting skeletal health: a 4-year longitudinal study assessing the effects of jumping on skeletal development in pre and circum pubertal children. Bone.

[4] Siris ES, Baim S, Nattiv A (2010). Primary care use of FRAX: absolute fracture risk assessment in postmenopausal women and older men. Postgrad Med.

[5] Daly RM (2007). The effect of exercise on bone mass and structural geometry during growth. Med Sport Sci.

[6] Hind K, Burrows M (2007). Weight-bearing exercise and bone mineral accrual in children and adolescents: a review of controlled trials. Bone.

[7] Fritz J, Rosengren BE, Dencker M, Karlsson C, Karlsson MK (2016). A seven-year physical activity intervention for children increased gains in bone mass and muscle strength. Acta Paediatr.

[8] Hughes JM, Petit MA (2010). Biological underpinnings of Frost’s mechanostat thresholds: the important role of osteocytes. J Musculoskelet Neuronal Interact.

[9] Macdonald HM, Kontulainen SA, Khan KM, McKay HA (2007). Is a school-based physical activity intervention effective for increasing tibial bone strength in boys and girls?. J Bone Miner Res.

[10] Macdonald HM, Cooper DM, McKay HA (2009). Anterior-posterior bending strength at the tibial shaft increases with physical activity in boys: evidence for non-uniform geometric adaptation. Osteoporosis Int.

[11] Rantalainen T, Nikander R, Heinonen A, Daly RM, Sievanen H (2011). An open source approach for regional cortical bone mineral density analysis. J Musculoskelet Neuronal Interact.

[12] Atkinson PJ, Weatherell JA (1967). Variation in the density of the femoral diaphysis with age. J Bone Joint Surg Br.

[13] Robling AG, Hinant FM, Burr DB, Turner CH (2002). Improved bone structure and strength after long-term mechanical loading is greatest if loading is separated into short bouts. J Bone Miner Res.

[14] Seeman E (2008). Structural basis of growth-related gain and age-related loss of bone strength. Rheumatology.

[15] Bousson V, Meunier A, Bergot C, Vicaut E, Rocha MA, Morais MH, Laval-Jeantet AM, Laredo JD (2001). Distribution of intracortical porosity in human midfemoral cortex by age and gender. J Bone Miner Res.

[16] Rantalainen T, Weeks BK, Nogueira RC, Beck BR (2015). Effects of bone-specific physical activity, gender and maturity on tibial cross-sectional bone material distribution: a cross-sectional pQCT comparison of children and young adults aged 5-29 years. Bone.

[17] Rantalainen T, Nikander R, Daly RM, Heinonen A, Sievanen H (2011). Exercise loading and cortical bone distribution at the tibial shaft. Bone.

[18] Duckham RL, Rantalainen T, Ducher G, Hill B, Telford RD, Telford RM, Daly RM (2016). Effects of habitual physical activity and fitness on tibial cortical bone mass, structure and mass distribution in pre-pubertal boys and girls: the look study. Calcif Tissue Int.

[19] Valdimarsson O, Linden C, Johnell O, Gardsell P, Karlsson MK (2006). Daily physical education in the school curriculum in prepubertal girls during 1 year is followed by an increase in bone mineral accrual and bone width–data from the prospective controlled Malmo pediatric osteoporosis prevention study. Calcif Tissue Int.

[20] Lofgren B, Daly RM, Nilsson JA, Dencker M, Karlsson MK (2013). An increase in school-based physical education increases muscle strength in children. Med Sci Sports Exerc.

[21] Linden C, Ahlborg HG, Besjakov J, Gardsell P, Karlsson MK (2006). A school curriculum-based exercise program increases bone mineral accrual and bone size in prepubertal girls: two-year data from the pediatric osteoporosis prevention (POP) study. J Bone Miner Res.

[22] Sundberg M, Gardsell P, Johnell O, Karlsson MK, Ornstein E, Sandstedt B, Sernbo I (2001). Peripubertal moderate exercise increases bone mass in boys but not in girls: a population-based intervention study. Osteoporosis Int.

[23] Sundberg M, Gardsell P, Johnell O, Karlsson MK, Ornstein E, Sandstedt B, Sernbo I (2002). Physical activity increases bone size in prepubertal boys and bone mass in prepubertal girls: a combined cross-sectional and 3-year longitudinal study. Calcif Tissue Int.

[24] Duppe H, Gardsell P, Johnell O, Nilsson BE, Ringsberg K (1997). Bone mineral density, muscle strength and physical activity. A population-based study of 332 subjects aged 15-42 years. Acta Orthop Scand.

[25] Schoenau E, Neu CM, Rauch F, Manz F (2001). The development of bone strength at the proximal radius during childhood and adolescence. J Clin Endocrinol Metab.

[26] Tan VP, Macdonald HM, Kim S, Nettlefold L, Gabel L, Ashe MC, McKay HA (2014). Influence of physical activity on bone strength in children and adolescents: a systematic review and narrative synthesis. J Bone Miner Res.

[27] Daly RM, Ducher G, Hill B, Telford RM, Eser P, Naughton G, Seibel MJ, Telford RD (2016). Effects of a specialist-led, school physical education program on bone mass, structure, and strength in primary school children: a 4-year cluster randomized controlled trial. J Bone Miner Res.

[28] Anliker E, Dick C, Rawer R, Toigo M (2012). Effects of jumping exercise on maximum ground reaction force and bone in 8- to 12-year-old boys and girls: a 9-month randomized controlled trial. J Musculoskelet Neuronal Interact.

[29] Heinonen A, Sievanen H, Kannus P, Oja P, Pasanen M, Vuori I (2000). High-impact exercise and bones of growing girls: a 9-month controlled trial. Osteoporosis Int.

[30] Bradney M, Pearce G, Naughton G, Sullivan C, Bass S, Beck T, Carlson J, Seeman E (1998). Moderate exercise during growth in prepubertal boys: changes in bone mass, size, volumetric density, and bone strength: a controlled prospective study. J Bone Miner Res.

[31] Peterman MM, Hamel AJ, Cavanagh PR, Piazza SJ, Sharkey NA (2001). In vitro modeling of human tibial strains during exercise in micro-gravity. J Biomech.

[32] Bass SL, Saxon L, Daly RM, Turner CH, Robling AG, Seeman E, Stuckey S (2002). The effect of mechanical loading on the size and shape of bone in pre-, peri-, and postpubertal girls: a study in tennis players. J Bone Miner Res.

[33] Ducher G, Daly RM, Bass SL (2009). Effects of repetitive loading on bone mass and geometry in young male tennis players: a quantitative study using MRI. J Bone Miner Res.

[34] Hsieh YF, Turner CH (2001). Effects of loading frequency on mechanically induced bone formation. J Bone Miner Res.

[35] Skerry TM (2006). One mechanostat or many? Modifications of the site-specific response of bone to mechanical loading by nature and nurture. J Musculoskelet Neuronal Interact.

[36] Wehner T, Claes L, Simon U (2009). Internal loads in the human tibia during gait. Clin Biomech.

[37] Ward KA, Roberts SA, Adams JE, Mughal MZ (2005). Bone geometry and density in the skeleton of pre-pubertal gymnasts and school children. Bone.

[38] Bousson V, Bergot C, Meunier A, Barbot F, Parlier-Cuau C, Laval-Jeantet AM, Laredo JD (2000). CT of the middiaphyseal femur: cortical bone mineral density and relation to porosity. Radiology.

[39] Farr JN, Blew RM, Lee VR, Lohman TG, Going SB (2011). Associations of physical activity duration, frequency, and load with volumetric BMD, geometry, and bone strength in young girls. Osteoporosis Int.

[40] Sherar LB, Baxter-Jones AD, Mirwald RL (2004). Limitations to the use of secondary sex characteristics for gender comparisons. Ann Hum Biol.

[41] Rasmussen AR, Wohlfahrt-Veje C, Tefre de Renzy-Martin K, Hagen CP, Tinggaard J, Mouritsen A, Mieritz MG, Main KM (2015). Validity of self-assessment of pubertal maturation. Pediatrics.

[42] Rabbani A, Noorian S, Fallah JS, Setoudeh A, Sayarifard F, Abbasi F (2013). Reliability of pubertal self assessment method: an Iranian study. Iran J Pediatr.

[43] Duke PM, Litt IF, Gross RT (1980). Adolescents’ self-assessment of sexual maturation. Pediatrics.

[44] Chan NP, Sung RY, Kong AP, Goggins WB, So HK, Nelson EA (2008). Reliability of pubertal self-assessment in Hong Kong Chinese children. J Paediatr Child Health.

